# Evidence through Thermal Analysis of Retro Diels-Alder Reaction in Model Networks Based on Anthracene Modified Polyester Resins

**DOI:** 10.3390/polym15194028

**Published:** 2023-10-09

**Authors:** Daniela Ionita, Mariana Cristea, Constantin Gaina, Mihaela Silion, Bogdan C. Simionescu

**Affiliations:** “Petru Poni” Institute of Macromolecular Chemistry, Aleea Grigore Ghica 41A, 700487 Iasi, Romania; ionita.daniela@icmpp.ro (D.I.); gcost@icmpp.ro (C.G.); silion.mihaela@icmpp.ro (M.S.); bcsimion@icmpp.ro (B.C.S.)

**Keywords:** Diels–Alder reaction, maleimide polyester resins, sample-controlled thermal analysis, anthracene Diels–Alder adducts

## Abstract

The present work is focused on polyester resins obtained from the diglycidyl ether of bisphenol A and anthracene modified 5-maleimidoisophthalic acid. Because the maleimide-anthracene Diels–Alder (DA) adduct is stable at high temperatures, it is considered a good option for high performance polymers. However, the information related to the retroDA reaction for this type of adduct is sometimes incoherent. A detailed thermal study (conventional TGA, HiRes TGA, MTGA, DSC, MDSC) was performed in order to establish whether the rDA reaction can be revealed for this type of anthracene modified polyester resins. The TGA method confirmed the cleavage of the anthracene–maleimide DA adduct, while the DSC demonstrated the presence of anthracene in the system. At high temperatures, unprotected maleimide homopolymerizes and/or reacts with allyl groups according to the –ene reaction. Therefore, the thermal DA reaction is not displayed anymore upon the subsequent cooling, and the glass transition region is registered at a higher temperature range during the second heating. The use of sample-controlled thermal analysis (HiRes TGA) and MTGA improved the TGA result; however, it was not possible to separate the very complex degradation processes that are interconnected.

## 1. Introduction

The notion of self-healing—simply defined as the potential of a polymeric material to be mended after damage, through physical or chemical strategies [1]—was unconceivable a few decades ago for epoxy- and maleimide-based polymers [2,3,4,5]. These classes of polymers exhibit high performance characteristics, though they come along with an intrinsic brittleness. A great deal of effort was directed towards finding the optimum equilibrium between the desired thermo-mechanical properties and the toughness. From the moment when the concept of dynamic covalent chemistry was introduced by Lehn in his landmark article, maleimide-based polymers were very soon largely investigated for their ability to function as a dienophile in the Diels–Alder (DA) reaction, as a modality for self-healing [6,7,8]. The temperature is the stimulus for the retrodienic reaction, which might be reversible by cooling, which triggers the repair of the polymeric material. The furan/maleimide (diene/dienophile) association is by far the most investigated, comprising multitudinous structures under scrutiny [9,10,11,12,13,14,15,16]. The reason for this interest lies in the approachability of the process, due to the fact that the furan/maleimide DA reaction takes place at room temperature and that the retro DA (rDA) reaction does not need excessive heating. The reported temperatures for the rDA reaction of furan/maleimide-based polymers do not exceed 150 °C. This fact can be an inconvenience for self-healing polymers that are intended for applications at high temperatures [17]. Anthracene can be an alternative for the diene in the case where the polymers have distinctive structures or where self-healing needs to be functional at elevated temperatures [18,19,20,21,22,23,24]. In the work of B. Gacel et al., graft copolymers were synthesized using anthracene-functionalized polystyrene and maleimido-functionalized poly(ethylene glycol) or poly(methyl methacrylate) [18], but only their glass transition temperatures were reported. In a very recent review, Briou at al. excellently systematized the issues related to the use of anthracene in DA reactions when it is connected to polymers: the lack of direct evidence of the rDA reaction and the inconsistency of the values reported as the rDA temperature [25]. Very frequently, the scientific literature cites the work of Grigoras and Colontin who probed the rDA reaction by thermogravimetric analysis (TGA) by heating the adduct obtained between the anthracene-based dienes and the telechelic dienophiles containing bismaleimide functions [26]. Shah et al. prepared side-chain-free highly aromatic polyimides containing anthracene via DA chemistry [19]. Actually, the diamine precursor was a DA adduct and was responsible for the formation of fully aromatic polymers. The rDA reaction temperature was determined by TGA above 215 °C. Recently, TGA was also used for this purpose in the case of anthracene/bismaleimide DA elastomers [27]. Specifically, anthracene moieties were attached to copolymers of ethylene, vinyl acetate, and glycidyl methacrylate (EVA-GMA copolymers). Covalent adaptable (reversible) networks were obtained by crosslinking with two dienophiles, (1,1′-(methylenedi-4,1-phenylene) bismaleimide and bifunctional 1,2,4-triazoline-3,5-dione), via DA chemistry. Aside from the DSC features, the TGA curves showed a substantial mass loss in the range 130 ÷ 330 °C, associated with the cleavage of the Diels–Alder adducts. Concerning the cleaving ability of polymers with anthracene and maleimide, a very important aspect was clearly underlined by Heo et al. [22]. They synthesized high thermally stable polyurethanes form isocyanate-terminated prepolymers and triethanol amines, with different ratios diisocyanate/triethanol amine. The prepolymers resulted through the addition of hexamethylene diisocyanate to pre-crosslinked adducts of 9-anthracenemethanol and N-(2-hydroxyethyl)-maleimide. Because of the high thermal stability of this type of polymer, the mechanical DA cleavage was also considered instead of the thermal rDA reaction. Another perspective is presented by Yoshie et al. in the case of networks with self-mending ability, based on anthracene and maleimide DA adducts. The structures of these polymeric compounds—prepared starting from anthryl-telechelic poly(ethylene adipate) and tris-maleimide—were not stable at high temperature; therefore, mechanical stress was applied for the rDA reaction [20].

The aim of this paper was to probe, by thermal analysis, whether the thermal rDA reaction can be revealed for polyester resins obtained from diglycidyl ether of bisphenol A and anthracene modified 5-maleimidoisophthalic acid. In addition to conventional TGA and differential scanning calorimetry (DSC), sample-controlled thermal analysis (HiRes TGA), modulated TGA (MTGA), and modulated DSC (MDSC) will be considered. Peng et al. used MDSC in the study of four-armed cross-linked polymers obtained via furan/maleimide DA reaction to separate the glass transition region (reversing heat flow signal) and the rDA reaction (non-reversing heat flow signal) [28]. These two phenomena appeared to be overlapping in the total heat flow signal. In HiRes TGA, the heating profile is controlled by the feedback from the sample, such as the heating rate defined for the experiment decreasing consistently during the mass loss [29,30]. The general purpose of this mode of operation is to obtain a better resolution of thermal events. In MTGA, a modulated temperature profile is superposed on the conventional linear heating one [31]. The activation energy is obtained for the whole range of the experimental conversion in one run, and in much less time than the conventional procedures. These two TGA options (HiRes TGA and MTGA) have rarely been used for the investigation of polymers [32,33,34,35,36,37] and, as we are aware, there are no such results reported for Diels–Alder polymers.

## 2. Materials and Methods

### 2.1. Materials

Maleic anhydride (99%), 5-aminoisophtalic acid (94%), diglycidyl ether of bisphenol A (DGEBA, epoxide equivalent weight 188 g·eq.^−1^), acetic acid, benzyltriethylammonium chloride (TEBAC, 99%), anthracene, methanol, N-methylpyrrolidone (NMP), ethylene glycol butyl ether (BEG), 2-methoxyethanol, and methanol were purchased from Sigma Aldrich, Darmstadt, Germany. All reactants and solvents were analytical grade and were used as received.

### 2.2. Synthesis of Polyester Resins Derived from DGEBA and 5-Maleimidoisophtalic Acid (DGEBA-MIPA)

First, 5-maleimidoisophthalic acid (MIPA) was prepared as previously reported [38,39]. Briefly, a mixture of 5-aminoisophthalic acid (7 g), maleic anhydride (4 g), and 60% acetic acid was refluxed for 4 h. The obtained solid was washed with water and methanol and dried at 100 °C under vacuum.

**Scheme 1:**
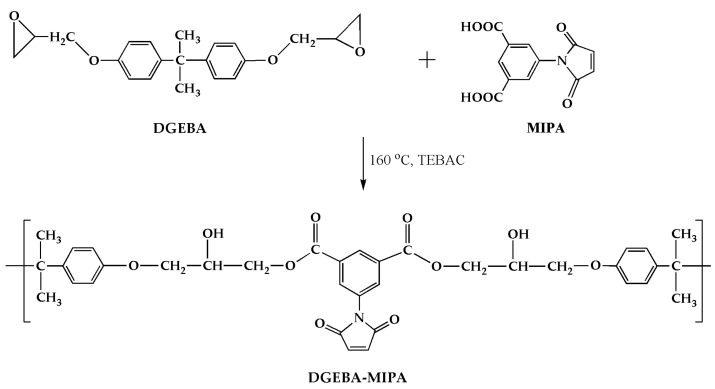
Synthesis of DGEBA-MIPA.

A mixture of DGEBA (3.0 g) and MIPA (2.06 g) dissolved in N-methylpyrrolidone (1/1 molar ratio, 15% solid mass) was stirred for 3 h at 160 °C, in the presence of benzyltriethylammonium chloride (Scheme 1). The solution was cast on a glass plate and the solvent was removed by heating at 120 °C for 6 h.

### 2.3. Synthesis of Polyester Resins Derived from DGEBA and 5-(9,10-Dihydro-9,10-ethanoanthracene-11,12-dicarboximido) Maleimidoisophtalic Acid (DGEBA-Anth)

Anthracene (1.8 g) was added to 5-maleimidoisophthalic acid (2.7 g) dissolved in 50 mL 2-methoxyethanol and refluxed for 3 h. After cooling and precipitation in water, the anthracene–maleimido anhydride diacid adduct (AMADA) was washed twice with methanol, filtered, and dried for 3 h at 90 °C.

**Scheme 2:**
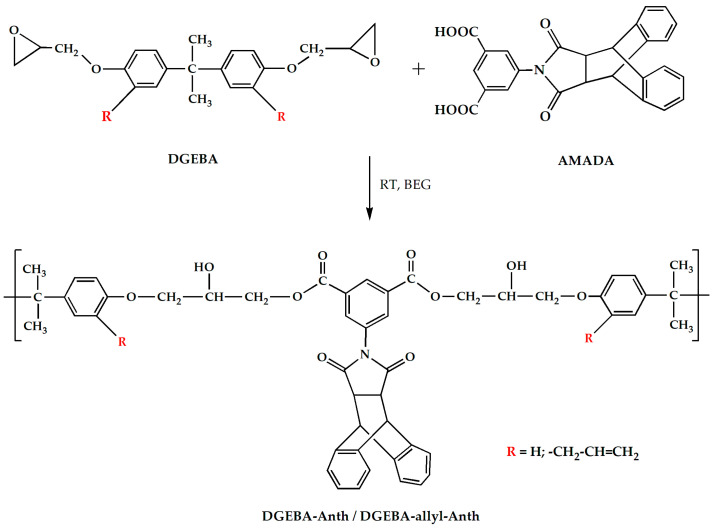
Synthesis of DGEBA-Anth (R: H) and DGEBA-allyl-Anth (R: -CH_2_-CH=CH_2_).

A quantity of 3 g DGEBA (188 g·eq.^−1^) was dissolved in BEG in the reaction vessel, at room temperature, under stirring. Separately, AMADA (2.8 g) was dissolved in BEG, under ultrasonication, for 10 min, and the solution was added to DGEBA, under stirring (Scheme 2). The reaction mixture was refluxed (170 °C) for 3 h. The final product was precipitated in methanol and dried at 100 °C for 6 h.

### 2.4. Synthesis of Polyester Resins Derived from Diglycidylether of o,o′-Diallyl Bisphenol A (DADGBPA) and 5-(9,10-Dihydro-9,10-ethanoanthracene-11,12-dicarboximido) Maleimidoisophtalic Acid (DGEBA-allyl-Anth)

The compound DADGBPA was prepared, as previously described [40,41], via o,o′-diallyl bisphenol A, by its reaction with epichlorohydrine and subsequent dehydrohalogenation. AMADA (5 g) dissolved in BEG was added to DADGBPA (4.44 g) dissolved in BEG and the reaction mixture was refluxed for 3 h in the presence of TEBAC (Scheme 2). The final product was precipitated in methanol and dried at 100 °C for 6 h.

### 2.5. Characterization

The Fourier transform infrared (FTIR) spectra were recorded on a Bruker Vertex 70 instrument (Bruker, Ettlingen, Germany), equipped with a Golden Gate single reflection ATR accessory. The spectra were measured in the range of 4000–600 cm^−1^, with a nominal resolution of 2 cm^−1^, after 32 scans.

The thermogravimetric analyses of the samples were performed on a Discovery TGA 5500 (TA Instruments, New Castle, DE, USA). The tests were conducted by using three heating rate algorithms: constant heating rate (20 °C/min) and dynamic heating rate (HiRes TGA and modulated approach-MTGA), from ambient temperature to 700 °C. The HiRes TGA experiments were performed at resolution (R) 3 and sensitivity (S) 1 and 2, using the constant heating rates of 20 °C/min and 50 °C/min. The MTGA experiments were performed with 2 °C/min, a modulation amplitude of ±5 °C, and a period of 200 s.

Differential scanning calorimetry (conventional—DSC and modulated—MDSC) was performed with a Discovery DSC 250 (TA Instruments, New Castle, DE, USA) under nitrogen atmosphere (50 mL/min). The heating–cooling–heating program with a heating rate of 20 °C/min was employed, unless otherwise specified. The MDSC analysis was carried out with 3 °C/min heating rate, modulation amplitude ±1 °C, and modulation period of 60 s.

The data acquisition and analysis for the mass spectrometry (MS) were performed with an Agilent 6500 Series Accurate-Mass Quadrupole Time-of-Flight (Q-TOF) LC/MS instrument (Agilent Technologies, Santa Clara, CA, USA). The DGEBA-allyl-Anth dissolved in DMF:methanol (9:1, *v*/*v*) was introduced into the electrospray source (ESI) via a syringe pump at a flow rate of 0.02 mL/min. All spectra were acquired in the range *m*/*z* 100–3000, in negative ion mode (ESI-), with a source temperature of 325 °C and a capillary voltage of 4.2 kV. Nitrogen (N_2_) was used as the drying/nebulizer gas, at 35 psi and a flow rate of 8 L/min. The mass scale was calibrated using the standard calibration procedure and the compounds were provided by the manufacturer. Data were collected and processed using Mass Hunter Workstation Software Data Acquisition for 6200/6500 series, v. B.07.00 (Agilent Technologies, Inc., Santa Clara, CA, USA).

## 3. Results and Discussion

During the synthesis of the epoxy resins containing maleimide groups (DGEBA-MIPA, Scheme 1), the formation of brittle high crosslinked compounds cannot be avoided, mainly because of the homopolymerization of the maleimide groups. The maleimide groups can be protected with anthracene through a DA reaction (Scheme 2, DGEBA-Anth). Moreover, the maleimide rigid systems can be toughened with reactive diluents [5,40,42,43], a procedure that was considered additionally for the synthesis of the DGEBA-allyl-Anth (Scheme 2) by using a diallyl bisphenol as reactant.

### 3.1. Structural Characterization of Polyester Resins

**Figure 1:**
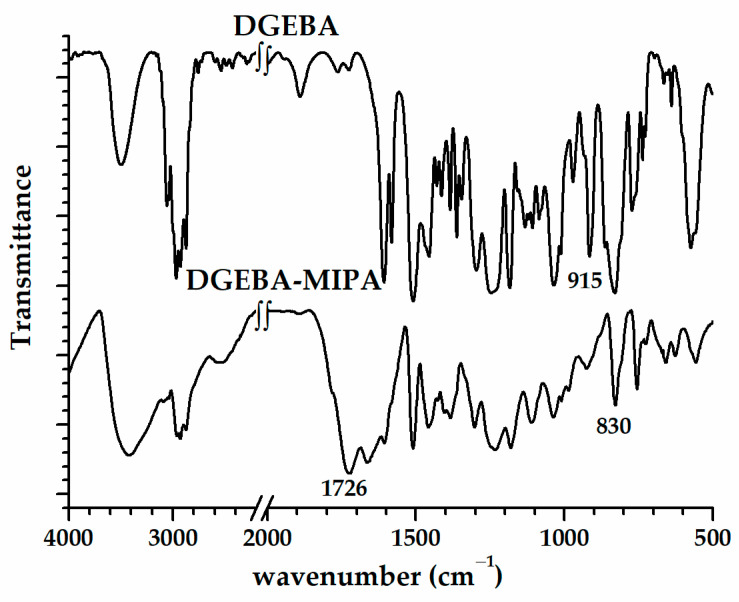
FTIR spectra of DGEBA and DGEBA-MIPA.

Figure 1 presents the FTIR spectra of DGEBA and DGEBA-MIPA comparatively.

The absence of the absorption peak of an oxirane ring (915 cm^−1^ in DGEBA) was the main indication of the occurrence of the addition reaction between the carboxylic acid groups of MIPA and the oxyrane groups of DGEBA [40,42,44]. The presence of the anhydride ring in the final resin is confirmed by the presence of the peaks associated with the carbonyl groups (1726 cm^−1^) and—C=C– linkage (830 cm^−1^), both belonging to the cyclic imide group [39,40,41,45].

**Figure 2:**
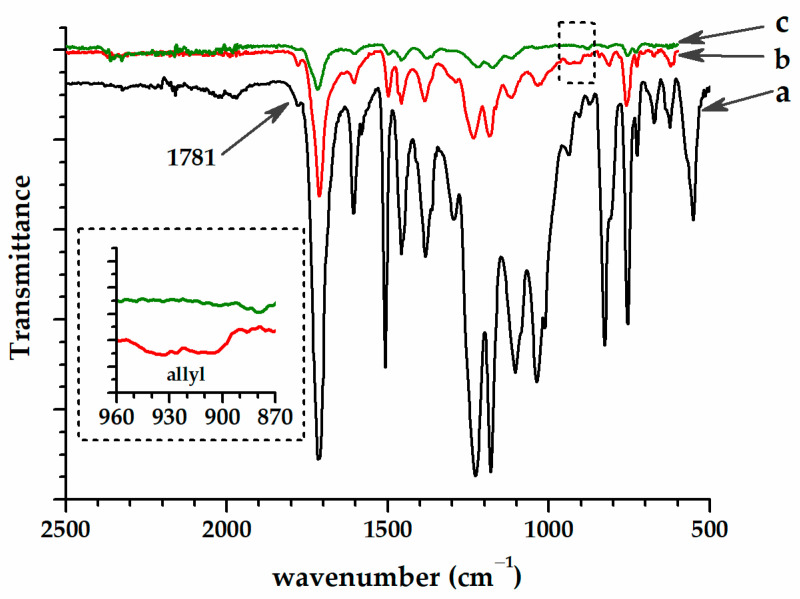
FTIR spectra of DGEBA-Anth (**a**), DGEBA-allyl-Anth (**b**), and DGEBA-allyl-Anth 300 (**c**). The inset figure details the signals of the allyl groups.

Figure 2 includes the FTIR spectra of the DGEBA-Anth and the DGEBA-allyl-Anth, as a resulted of the syntheses, and of one more sample cured at 300 °C (DGEBA-allyl-Anth 300).

The formation of the adduct is supported by the 1781 cm^−1^ peak found in both compounds [46,47,48]. The heating of the DGEBA-allyl-Anth sample at 300 °C prompted the deprotection of the maleimide groups—associated with the estompation of the adduct signal in the DGEBA-allyl-Anth 300—along with their cycloaddition to the allyl groups (DGEBA-allyl-Anth, 890–960 cm^−1^), which are no longer present in the DGEBA-allyl-Anth 300 (inset of Figure 2).

### 3.2. Thermogravimetric Analysis of Polyester Resins

**Figure 3:**
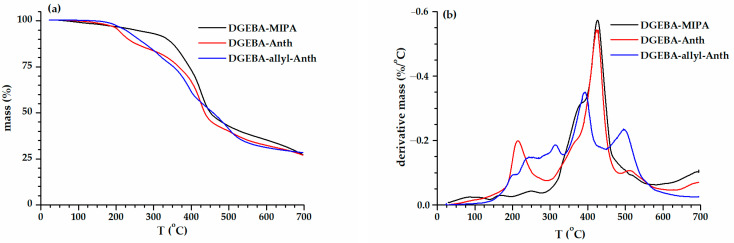
Conventional thermal degradation curves for DGEBA-MIPA, DGEBA-Anth, and DGEBA-allyl-Anth (20 °C/min, N_2_ environment): (**a**) TGA, (**b**) DTGA.

The results of the conventional TGA, expressed in the evolution of the sample mass and its temperature derivative (DTGA), are represented in Figure 3a,b. Also, Table 1 contains the main thermal characteristics of the samples of the DGEBA-MIPA, DGEBA-Anth, and DGEBA-allyl-Anth. The values of the onset degradation temperature are much lower for the samples with anthracene than for the sample DGEBA-MIPA (Table 1). The DTGA curve of the sample DGEBA-Anth exhibited a sharp peak centered at 215 °C, which can be associated with the cleavage of the Diels–Alder adduct maleimide-anthracene (Figure 3a).

In the case of the DGEBA-allyl-Anth, there is a succession of small DTGA peaks/shoulders (200 °C, 245 °C, 275 °C). They were connected with the deprotection of the maleimide groups, followed by the –ene reaction with allyl groups (Wagner-Juaregg intermediate) and the subsequent Diels–Alder reaction that generates a cyclic network structure [4,49]. The cleavage of the ether–ether linkage and the decomposition of succinimide groups are reflected in the main degradation range (350–450 °C). These two degradation steps are not well-separated for the DGEBA-MIPA and the DGEBA-Anth in the conventional TGA experiment. The DTGA peaks recorded around 500 °C on the conventional thermal degradation curves of the DGEBA-Anth and the DGEBA-allyl-Anth evidence the decomposition of the aromatic cycles.

**Figure 4:**
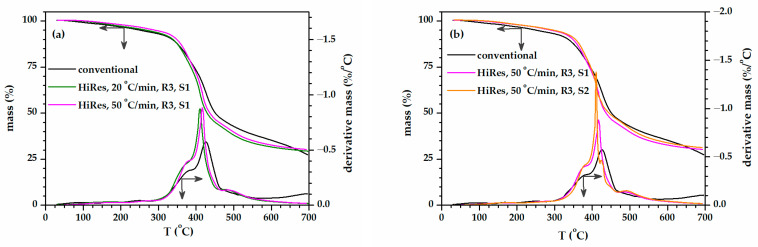
Conventional (20 ^o^C/min) and HiRes thermal degradation curves for DGEBA-MIPA (N_2_ environment): (**a**) the constant heating rates are different; (**b**) the sensitivities (S) are different.

Given the fact that the HiRes TGA approach could distinguish very close degradation peaks, the question is whether a better separation of the main degradation steps from the range 350–450 °C is possible. As a preliminary requirement, the experimental conditions (constant heating rate, resolution, and sensitivity) should be suitably chosen, so that the experimental time may be comparable with that of the conventional TGA experiment. Otherwise, kinetic effects deem the conventional TGA and HiRes TGA results inadequate for comparative discussions. The constant heating rate is the value of the heating rate in the absence of degradation events. As the balance detects degradation, the heating rate was implicitly decreased until no degradation was sensed. According to our practice, resolution 3 is the most adequate for our samples to fulfill this essential. Figure 4a includes the results of conventional TGA (heating rate: 20 °C/min) and HiRes TGA (constant heating rates: 20 and 50 °C/min, R3, and S1) for the DGEBA-MIPA sample. The two peaks shifted to a lower temperature and intensified in the HiRes experiment, but their resolution was not improved. The use of two different constant heating rates did not produce an important change in the result. The modification of sensitivity from 1 to 2 (Figure 4b) increased the sharpness of the main peak, without an improved differentiation of the peaks in the main degradation stage. Nevertheless, unlike conventional TGA (Figure 3b), the HiRes experiment also marks the DGEBA-MIPA for the degradation of the aromatic cycles (around 500 °C).

**Figure 5:**
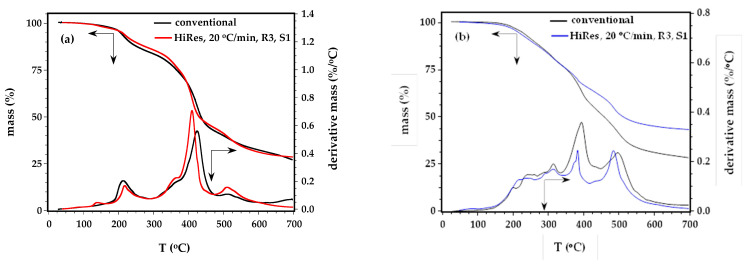
Conventional and HiRes thermal degradation curves for DGEBA-Anth (**a**) and DGEBA-allyl-Anth (**b**) (constant heating rate 20 °C/min, R3, S1, N_2_ environment).

Similarly, when the HiRes approach was applied to the DGEBA-Anth and the DGEBA-allyl-Anth (Figure 5a,b), there were some ameliorations in the clarity of the degradation steps, but the overlapping degradation phenomena were not better separated.

**Figure 6:**
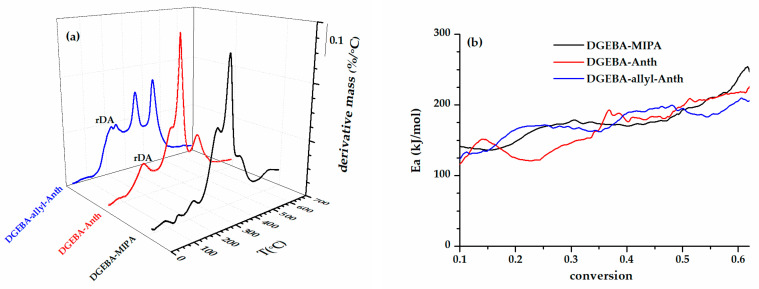
Thermal degradation curves obtained by MTGA (**a**) and the associated activation energy (**b**) all along the experiments.

In the MTGA experiments (Figure 6a), the degradation steps discussed before were more obvious, including the rDA reaction. However, it is worth recalling that the heating rate was much lower (2 °C/min). The MTGA experiment lasted 340 min, as compared to 34 min (conventional TGA) and 47 min (HiRes TGA, R3, S1). The variation in the activation energy with the conversion, registered in real time, shows a peak at 0.15 conversion (DGEBA-Anth) due to the cleavage of the maleimide–anthracene adduct. Generally, it had an increasing trend with the conversion, with values between 100 and 200 kJ/mol.

### 3.3. Differential Scanning Calorimetry Analysis of Polyester Resins

**Figure 7:**
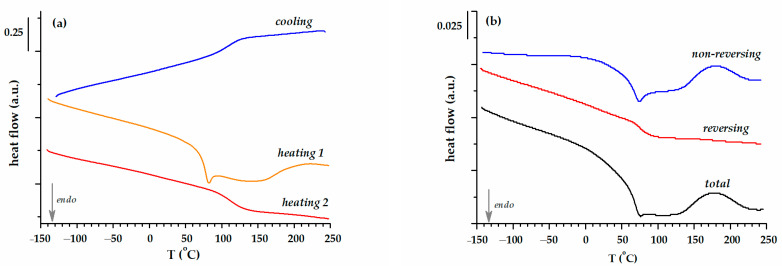
The result of DSC scans (heating 1–cooling–heating 2, 20 °C/min) (**a**) and MDSC scans (3 °C/min) (**b**) for DGEBA-MIPA.

Figure 7a displays the DSC curves (first heating-cooling-second heating) of the DGEBA-MIPA. In the first heating step, the glass transition region (onset of the glass transition temperature—T_g_: 70.1 °C) was overlapped with a stress relaxation. The presence of an exothermal signal with the middle at 220 °C (±0.25 °C) is an indication of the maleimide groups homopolymerization. As the result of the formation of a crosslinked network, the glass transition region was shifted to higher temperatures in the cooling step (midpoint at 109.3 ± 0.25 °C) and in the second heating step (midpoint: 113.7 ± 0.25 °C). A particular feature of MDSC is the possibility to separate the glass transition and the enthalpic relaxation in two separate signals, reversible and non-reversible [50]. Therefore, the T_g_ of the initial DGEBA-MIPA was determined in a MDSC experiment as 53.9 °C (±0.25 °C) (Figure 7b). This value is smaller than the onset T_g_ determined in conventional DSC, because the heating rate was much lower (MDSC: 2 °C/min vs. DSC: 20 °C/min). Also, the exothermal peak associated with the homopolymerization of the maleimide groups was shifted to lower temperatures. To avoid crosslinking, the maleimide groups were protected with anthracene moieties (DGEBA-Anth and DGEBA-allyl-Anth).

**Figure 8:**
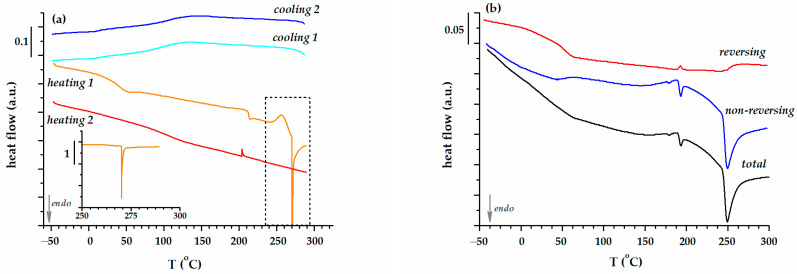
The result of DSC scans (heating 1,2 with 5 °C/min–cooling 1,2 with 10 °C/min) (**a**) and MDSC scans (3 °C/min) (**b**) for DGEBA-Anth. The inset of Figure 8a details the endothermal signal of anthracene.

The most prominent detail of the conventional DSC scan of the DGEBA-Anth is the appearance of the sharp endothermic peak in the first heating step (Figure 8a), at 270 °C. More than likely, the endothermic peak confirms the presence of the anthracene in the system [51], as a consequence of the deprotection of the maleimide groups (rDA reaction). This peak was displayed only when working with a hermetic crucible and at the heating rate of 5 °C/min. Some unprotected maleimide groups still exist in the initial sample, because the glass transition region was shifted to a higher temperature in the second heating step. No exothermic signal was registered in the cooling step as evidence for another Diels–Alder reaction. The endothermic peak associated with anthracene broadens in MDSC (Figure 8b), likely for kinetic reasons.

**Figure 9:**
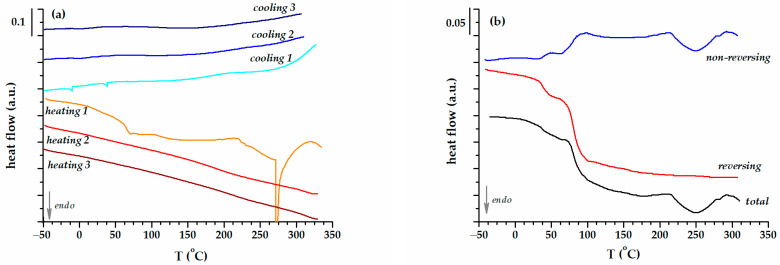
The result of DSC scans (heating 1,2,3 with 5 °C/min–cooling 1,2,3 with 10 °C/min) (**a**) and MDSC scans (3 °C/min) (**b**) for DGEBA-allyl-Anth.

The presence of anthracene in the system was also confirmed by the DSC scans of the DGEBA-allyl-Anth performed in a hermetic crucible (Figure 9a). It is worth noting that MDSC put forward two consecutive glass transition regions between 30 and 100 °C. This observation suggests that not all the maleimide groups reacted with anthracene. The endothermic peak associated with anthracene cleavage is broader than in the DGEBA-MIPA and DGEBA-Anth. As the anthracene is released, the maleimide groups can react with the allyl groups (-ene reaction via Wagner-Juaregg intermediate), and the viscosity of the product growths and the thermal transfer is delayed.

**Figure 10:**
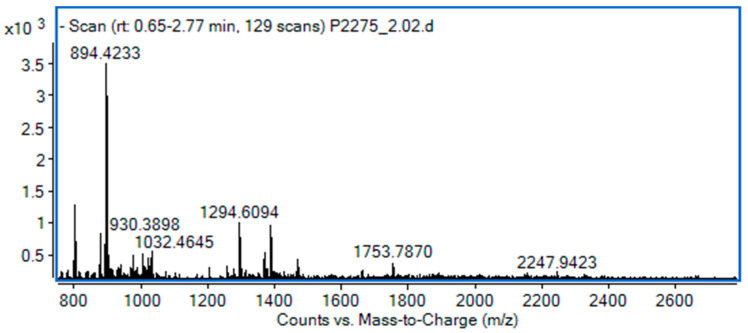
Electrospray ionization mass spectrometry (ESI-MS) spectrum of DGEBA-allyl-Anth.

The ESI-MS allows for the direct analysis of sample solutions; however, the size of the peaks do not offer a quantitative evaluation of the data, but only qualitative [52,53]. The method was employed for compounds synthesized via Diels–Alder chemistry in order to track the progress of the transformation [54,55]. Nebhani and Barner-Kowollik investigated a diene–dienophile pair composed of anthracenyl capped polyethylene glycols and fullerenes [56]. Table 2 includes some of the species that were identified by means of mass spectrometry for the sample DGEBA-allyl-Anth (Figure 10).

**Scheme 3:**
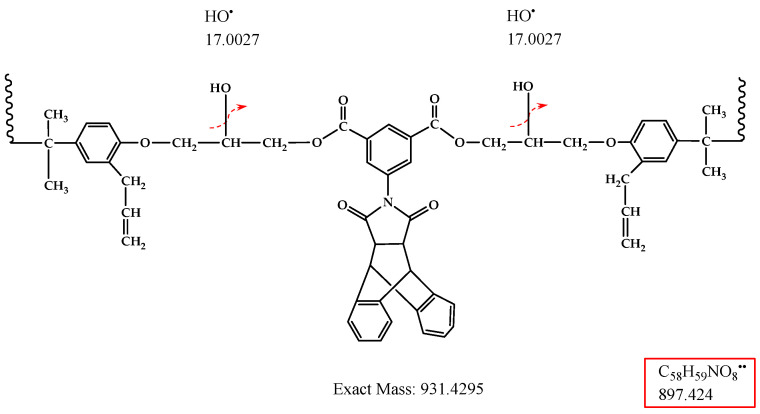
Structure of F1.

**Scheme 4:**
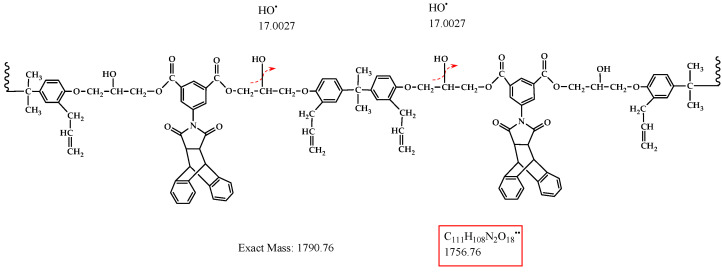
Structure of F2.

In the negative ESI-MS spectrum of the DGEBA-allyl-Anth (Figure 10) the most intense peak (*m*/*z* 896.45) is assigned to the fragment [F1–2OH–H]^−^, which arises from the loss of two hydroxyl groups of the [F1–H]^−^ species (*m*/*z* 930.39) (Scheme 3). The signal *m*/*z* 1755.76 can be attributed to the species [F2-2OH-H]^−^ (Scheme 4).

Moreover, double charged ions (2-) were also detected in the mass spectrum. The species [F2-2H]^2−^ and [F4-2H]^2−^ correspond to the molecular segments with 2 and 4 anthracene-protected maleimide groups, respectively (Scheme 3 and Scheme 4).

The double charged ions (2-) detected in the mass spectrum (*m*/*z* > 2000) suggest that the molecular structures of the species associated with the sample DGEBA-allyl-Ant may include 5 anthracene-protected maleimide groups (around 4500 Da).

The MS results are consistent with the prevalence of the maleimide groups protected with anthracene in the DGEBA-allyl-Anth sample. Nevertheless, after the thermal rDA reaction that takes place in the DSC oven, the unprotected maleimide is prone to homopolymerization or –ene reaction at high temperatures, with the formation of complex networks. The DA reaction was longer displayed on cooling and the glass transition region was shifted to higher temperatures during the next heating (Figure 9a).

## 4. Conclusions

In this study we prepared polyester resins based on DGEBA and allyl-modified DGEBA. The maleimide groups were protected with anthracene. The TGA investigations (conventional, HiRes TGA, and MTGA) confirmed the cleavage of the maleimide–anthracene adduct at 215 °C for the DGEBA-Anth and 200 °C for the DGEBA-allyl-Anth. The rDA was also sustained by the DSC sharp endothermic signal that is associated with the presence of the anthracene in the system after the break of the DA adduct. However, the DA reaction was not evidenced on the subsequent cooling. The preponderance of the species with anthracene-protected maleimide groups revealed by MS-ESI in the case of the DGEBA-allyl-Anth confirms the narrowness of the secondary reaction (maleimide homopolymerization, -ene reaction) during synthesis. However, the thermal rDA in the TGA and DSC instruments triggers the secondary reactions as the maleimide is no longer protected. The HiRes TGA performed on the samples improved the TGA result by featuring new degradation steps like aromatics but did not completely separate the main degradation processes because of the complexity of the decomposition reactions. The activation energy had an increasing trend with the conversion (100–200 kJ/mol) and a maximum was registered when the DA adduct separated.

## Figures and Tables

**Table 1:** The main thermal degradation characteristics of the investigated polyesters, from the conventional TGA results (heating rate 20 °C/min).

Sample	T_deg onset_^(1)^ (°C)	T_10_ ^(2)^ (°C)	Residue ^(3)^ (%)
DGEBA-MIPA	352.0	336.4	29.5
DGEBA-Anth	193.7	232.6	28.6
DGEBA-allyl-Anth	198.5	260.0	28.9

^(1)^ The temperatures of the degradation onset evaluated with the software TRIOS 5.0 (TA Instruments, New Castle, DE, USA). The standard error is ±0.33 °C. ^(2)^ The temperature associated with 10% of the mass loss. The standard error is ±0.35 °C. ^(3)^ The residual mass at 675 °C.

**Table 2:** Negative ion electrospray ionization mass spectrometry (ESI-MS) data for the sample DGEBA-allyl-Anth.

z	*m*/*z* Found	*m*/*z* Calculated	Species Assigned ^(1)^	M
2	894.42	894.37	[F2–2H]^2−^	1790.84
1	896.45	896.42	[F1–2OH–H]^−^	870.42
1	930.39	930.42	[F1–H]^−^	930.42
2	1753.78	1753.71	[F4–2H]^2−^	3509.56
1	1755.76	1775.74	[F2–2OH–H]^−^	1756.76

^(1)^ The structures are presented in Scheme 3 and Scheme 4.

## Data Availability

The data presented in this study are available on request from the corresponding author.
